# Scanning Techniques for Nanobioconjugates of Carbon Nanotubes

**DOI:** 10.1155/2018/6254692

**Published:** 2018-06-13

**Authors:** Kazuo Umemura, Shizuma Sato

**Affiliations:** Department of Physics, Tokyo University of Science, 1-3 Kagurazaka, Shinjuku, Tokyo 1628601, Japan

## Abstract

Nanobioconjugates using carbon nanotubes (CNTs) are attractive and promising hybrid materials. Various biological applications using the CNT nanobioconjugates, for example, drug delivery systems and nanobiosensors, have been proposed by many authors. Scanning techniques such as scanning electron microscopy (SEM) and scanning probe microscopy (SPM) have advantages to characterize the CNT nanobioconjugates under various conditions, for example, isolated conjugates, conjugates in thin films, and conjugates in living cells. In this review article, almost 300 papers are categorized based on types of CNT applications, and various scanning data are introduced to illuminate merits of scanning techniques.

## 1. Introduction

### 1.1. Nanobioconjugate Applications of Carbon Nanotubes

Carbon nanotubes (CNTs) are promising nanomaterials that have extraordinary structures and properties [1–7]. The robust and flexible structures of CNTs may allow the fabrication of various nanoarchitectures. Furthermore, the unique electrical and optical properties of CNTs may permit their use in various applications, such as nanosensors.

One of the important technical milestones for CNT nanotechnology is the separation of CNTs. CNTs synthesized from amorphous carbon have various lengths, diameters, and number of layers. The latter include single-walled CNTs (SWNTs), double-walled CNTs (DWNTs), and multiwalled CNTs (MWNTs). Furthermore, CNTs with various chiralities can be produced. Since the physicochemical properties of CNTs vary according to these factors, methods of isolating single-chirality CNTs have been proposed [8–13].

Among the diverse possible applications of CNTs, biological applications are important [14–35]. Biological applications require soluble CNTs; they often involve aqueous solutions. “Wrapping” techniques are popular approaches to solubilize CNTs [23, 26, 36–52]. When CNT powder is added to a surfactant solution followed by sonication, CNT bundles form and each bundle will become wrapped with surfactant molecules. In addition to surfactants, which were used to first demonstrate the feasibility of wrapping, various other organic molecules including DNA and protein molecules have also been successfully used to wrap CNTs [53–67]. Advantages of the use of DNA molecules have been described by several authors. For example, DNA and SWNTs are specifically related to DNA sequence and CNT chirality [66]. The authors suggested that (TCC)_10_, (TGA)_10_, and (CCA)_10_ have an avid affinity for (9, 1) SWNTs.

Scanning techniques are a powerful means to characterize the structures and physicochemical properties of CNTs and nanobioconjugates of CNTs, such as hybrids of DNA and CNTs (DNA-CNT hybrids). In this paper, various scanning studies of DNA-CNT and other nanobioconjugates of CNTs are categorized based on the types of biological applications. We previously published a review article summarizing scanning probe microscopy (SPM) studies of DNA-CNT hybrids [68]. In that paper, we categorized references based on the types of SPMs. In the present review, the references are categorized based on research targets. This review also includes scanning electron microscopy (SEM) to provide a comprehensive overview of research scanning techniques. The advantages of the scanning techniques in each CNT application are highlighted.

## 2. Advantages of Scanning Techniques in Studying Nanobioconjugates of CNTs

**Figure 1:**
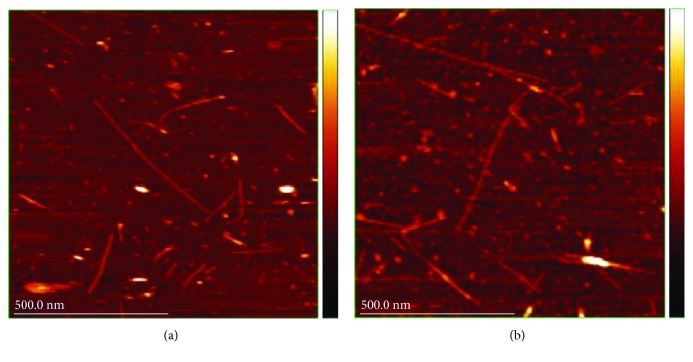
AFM images reported by Hayashida and Umemura. (a) Hybrids of ssDNA and SWNTs. (b) Hybrids of dsDNA and SWNTs. Observation was carried out in a buffer solution (reprinted from Hayashida and Umemura [69] with permission).

SPM and SEM are reasonable approaches to characterize nanobioconjugates with CNTs, as is transmission electron microscopy (TEM). Among the SPM techniques, atomic force microscopy (AFM) is frequently employed to obtain topographical information of CNT nanobioconjugates.  Figure 1 provides example AFM topographs of DNA-SWNTs hybrids in aqueous solution [69]. AFM imaging can clearly reveal rod-like structures of CNTs without complicated sample preparations. The authors found that the heights of the observed hybrids fluctuated according to environmental conditions.

**Figure 2:**
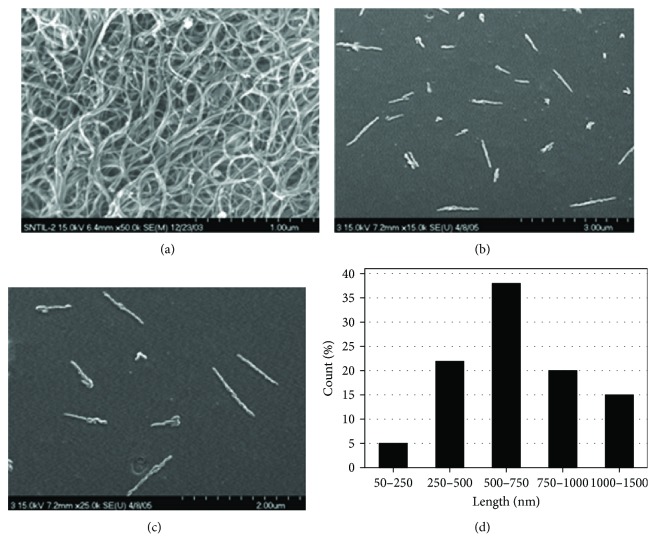
SEM images reported by Nepal et al. (a) An SEM image of SWNTs. (b and c) An SEM image of DNA-SWNTs. (d) Length distribution of DNA-SWNTs (reprinted from Nepal et al. [70] with permission).

Figure 2 shows SEM images of SWNTs and DNA-SWNT conjugates reported by Nepal et al. [70]. Bare SWNTs were observed as bundled structures (Figure 2(a)), but individual bundles were clearly resolved. In the case of DNA-SWNT conjugates, monodispersed SWNTs were also clearly evident. Furthermore, MWNTs were also observed by SEM and compared with SWNTs. A length distribution analysis determined that sample preparation procedures affected CNT length. TEM has also been used to visualize CNT nanobioconjugates. One study incorporated TEM along with AFM and SEM for similar samples [71].

Thin or thick films containing CNT nanobioconjugates are popular samples as well as isolated conjugates. Their surface morphologies have been mainly studied by SEM [25, 70, 72–82]). Scanning techniques have also been used to examine other various structures of CNT nanobioconjugates [83–91]. CNT nanobioconjugates have also been verified using energy dispersive X-ray spectrometry (EDS) [25, 91–95].

General characterization, which includes the use of scanning techniques, is a fundamental and crucial aspect of CNT-related studies. When bioconjugates of CNTs are isolated on a flat surface, the excellent high resolution afforded by SPM is a big advantage. When bioconjugates of CNTs are prepared as films, SEM and SPM can be selectively employed according to the roughness of the films.

## 3. CNT Nanobioconjugates for DNA Sensors

While one of the purposes of the aforementioned wrapping techniques is to solubilize CNTs, the wrapped structures also have potential value as nanobiodevices. This section described some notable scanning studies involving DNA wrapping.

**Figure 3:**
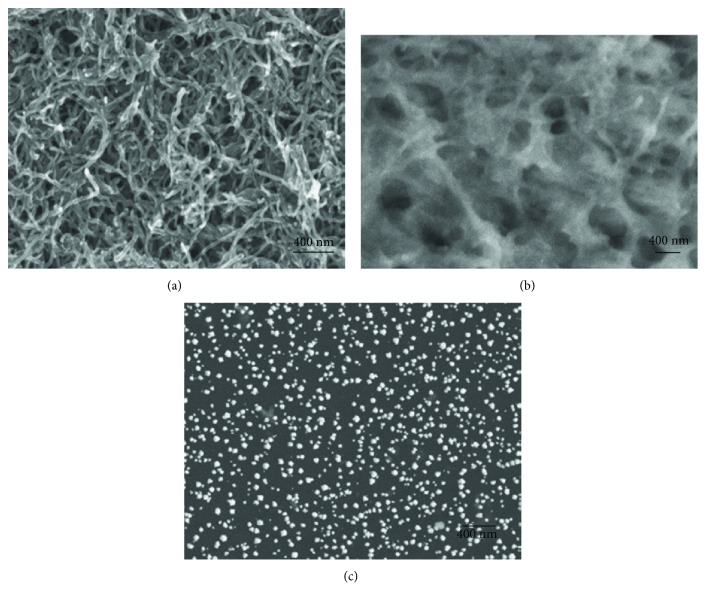
SEM images reported by Wang et al. (a) MWNTs/GCE. (b) PAbA/MWNTs/GCE. (c) Au nanoparticles/PABA/MWNTs/GCE (reprinted from Wang et al. [97] with permission).

An important application of CNTs for DNA studies is mismatch detection in the hybridization of DNA molecules [96–99]. One study reported on the fabrication of conjugates of MWNTs, gold nanoparticles (Au NPs), and poly(*p*-aminobenzoic acid) (PABA). After depositing the conjugates on a glassy carbon electrode (GCE) surface, single-stranded DNA (ssDNA) was attached to the conjugates by gold monosulfide bonding [98]. Hybridization between the attached ssDNA and a complementary ssDNA was detected by differential pulse voltammetry. The authors succeeded in detecting three-based mismatched ssDNA. Another study from the same research group expanded the method combining zinc oxide nanowires (ZnONWs) with gold nanoparticles and MWNTs and succeeded in detecting single-mismatched ssDNA [97]. SEM was employed to characterize the functionalized GCE surfaces. Figures 3(a)–3(c) show SEM images of MWNTs/GCE, PABA/MWNTs/GCE, and Au NPs/PABA/MWNTs/GCE, respectively [98]. Although the surface conditions were different among the three samples, surface structures were well characterized by SEM. In particular, distribution of Au NPs on the GCE was clearly visualized. SEM proved advantageous for these samples because of the large depth of field.

**Figure 4:**
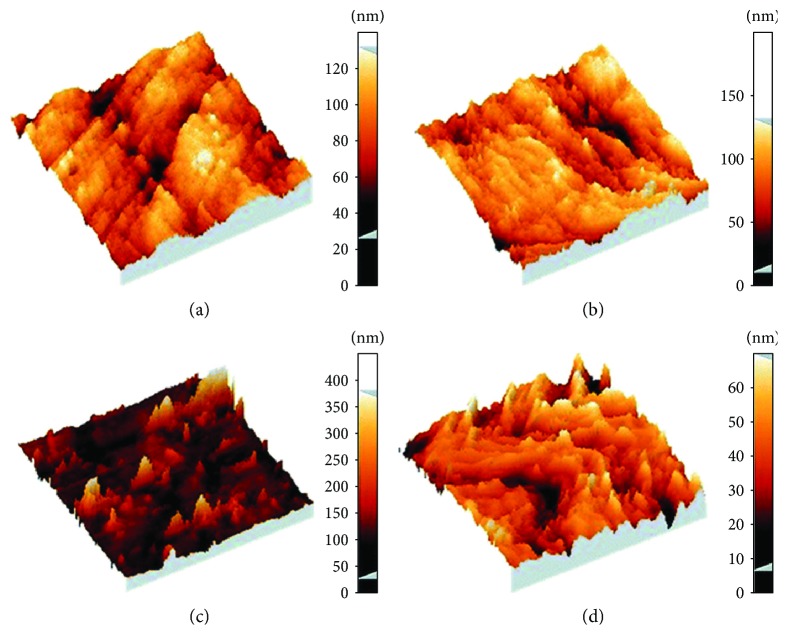
AFM images of functionalized GCE surfaces for DNA mismatch detection. (a) Oxidized bare GCE. (b and d) DNA/oxidized GCE. (c) MWNTs/DNA/oxidized GCE. Scan sizes: 5 × 5 *μ*m in (a), (b), and (c). 1 × 1 μm in (d) (reprinted from Shahrokhian et al. [96] with permission).

Mismatch detection on GCE has also been accomplished by combining DNA, MWNTs, and [Fe(CN)_6_]^3−/4−^ [96]. The authors used AFM to characterize the functionalized GCE surfaces (Figure 4).

It is simple to attach DNA molecules to CNT surfaces. Thus, CNTs could potentially be used as DNA sensors. When DNA hybridization is detected electrically, CNT nanobioconjugates for DNA detection are deposited on electrode surfaces. Electrode surfaces are not transparent in many cases. Thus, scanning techniques have obvious advantages over TEM and usual optical microscopes. Compared with SEM images, AFM has the advantage of enabling quantitative information concerning height. In contrast, when electrode surfaces are very rough, SEM is preferred.

Other studies have optically detected DNA hybridization using CNT nanobioconjugates. In this case, isolated DNA-CNT in suspension was used, rather than films on solid electrode surfaces [100–107]. Single-mismatch detection can also be done optically. Optical responses of CNTs, such as absorption and generation of photoluminescence spectra, can be used to detect DNA hybridization. Suspended DNA-CNT conjugates can be deposited on flat surfaces such as a cleaved mica surface for AFM observation.

In the future, direct detection of DNA associated with CNTs could be possible using scanning tunneling microscopy (STM). STM is a SPM-related technique that detects minute electrical signals, such as tunneling currents, between a conductive probe and a sample surface, as does AFM detects forces between a probe and a sample [108, 109]. In these electrical and optical detection techniques, plentiful CNTs are necessary for DNA detection. However, if STM could be adapted for this purpose, a single CNT might be sufficient to detect a single DNA molecule. Detection of DNA reaction with single DNA pairs with a single CNT is the dream of a single-molecule DNA sensor.

## 4. CNT Nanobioconjugates as Molecular Sensors

**Figure 5:**
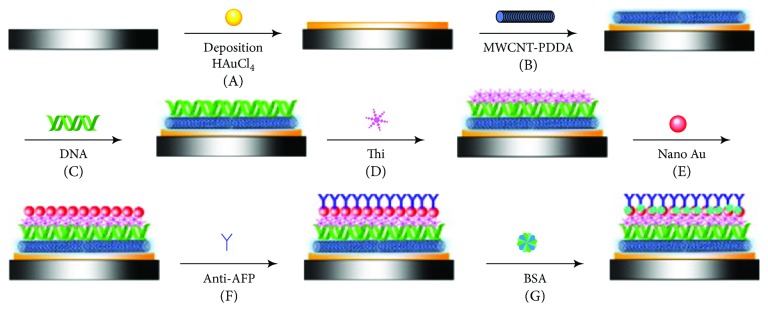
Schematic view of the fabrication process of an immunosensor reported by Ran et al. (A) Deposition of Au nanoparticles. (B) Coating of MWNTs-PDDA layer. (C) Immobilization of DNA film. (D) Formation of thionine layer. (E) Assembly of gold nanoparticles. (F) Anti-AFP loading. (G) BSA blocking (reprinted from Ran et al. [127] with permission).

In addition to the detection of DNA hybridization, CNT can detect various biological reactions [94, 96–99, 110–133]). One study described the fabrication of an immunosensor using CNTs (Figure 5) [127]. The authors immobilized MWNTs dispersed with poly(diallydimethlammonium chloride) (PDDA) on an Au nanofilm that had been electrochemically deposited on GCE. DNA and thionine were attached to the functionalized GCE. After depositing Au NPs on the thionine surface, alpha-fetoprotein (AFP) antibody was immobilized on the NPs. The fabricated GCE was available to electrochemically detect AFP using an immunoreaction between the AFP and AFP antibody. SEM observation of the GCE surfaces at each functionalization step was effective to verify their samples.

In another study, a lactate biosensor was created by combining a conductive polymer (poly-5,2′-5′,2^″^-ter-thiophene-3′-carboxylic acid; pTTCA) and MWNTs Rahman et al. [126]. After depositing pTTCA/MWNT films on a gold electrode, lactate dehydrogenase and the oxidized form of nicotinamide adenine dinucleotide (NAD^+^) were immobilized on the film. SEM was used to characterize the functionalized surfaces. Another recent study from the same laboratory demonstrated uric acid detection by this functionalized electrode [94].

A unique new method for sensor applications has been proposed [80, 124]. The authors deposited DNA-CNT conjugates between two Au electrodes. Since DNA bases have specific affinity with ions, such as Hg(II), Cd(II), and Pb(II), the nanodevice could be utilized as an ionic sensor. SEM was a reasonable method to characterize structures of their nanodevices.

The mechanisms of the electrical and optical responses of CNTs are not fully understood. Experimental data from various biomolecules are important to establish CNT biosensor applications. In this context, sample verification by scanning techniques is obviously important and systematic accumulation of data under similar experimental conditions is expected. The current reality is that studies are conducted using various CNT powders. Furthermore, CNTs with the same product number purchased from the same company can display differences in composition between lots. Hybridization of CNTs and biomolecules can also vary among researchers. Thus, the direct comparison of data obtained by different research groups is difficult.

## 5. Nanobioconjugates for Cell Researches

The use of CNT nanobioconjugates to study living cells is growing in popularity ([134–139] [28, 85, 140–167]). Scanning techniques are important and powerful tools in verifying samples.

**Figure 6:**
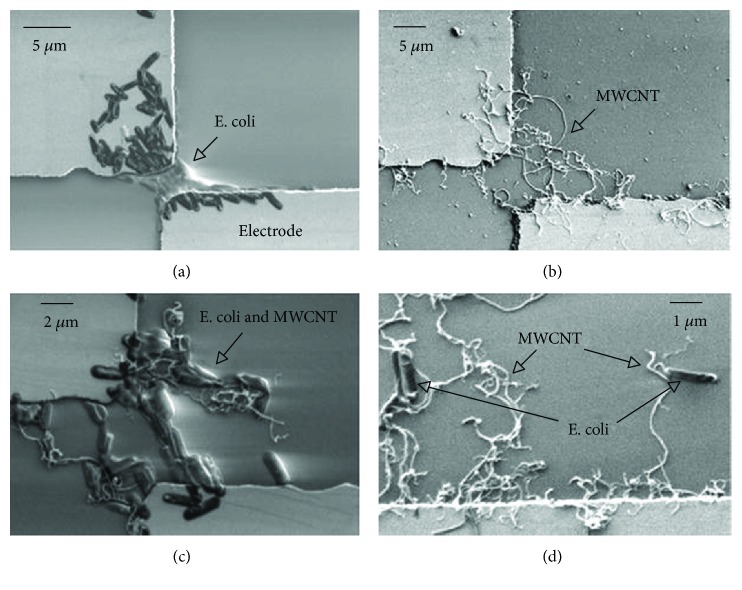
SEM images of trapped *E. coli* cells and MWNTs between two microelectrodes by DEP force. (a) Trapped cells. (b) Trapped MWNTs. (c) Trapped cells and MWNTs. (d) Cells trapped at the tip of MWNTs (reprinted from Suehiro et al. [157] with permission).

One of the important applications is CNT-mediated drug delivery [155, 161, 168–189]. The fabrication of bio/nanointerfaces of *Escherichia coli* and MWNTs has been described Suehiro et al. [157]. In the unique approach, a mixture of *E. coli* and solubilized MWNTs was deposited between two microelectrodes. *E. coli* was trapped by the MWNTs by dielectrophoresis force. SEM images clearly visualized the bacteria trapped between the electrodes (Figure 6).

For use in drug delivery, it is crucial to demonstrate that CNTs are not toxic. Many studies that did not utilize scanning techniques have intensively studied the toxicity of CNTs ([135, 169, 190–215]. The use of scanning techniques has proven advantageous for toxicity studies [85, 214, 216–219]. As one example, the effects of MWNTs on human lung epithelial cells evaluated using SEM and other assessments revealed MWNT-mediated cytotoxicity and genotoxicity [135].

**Figure 7:**
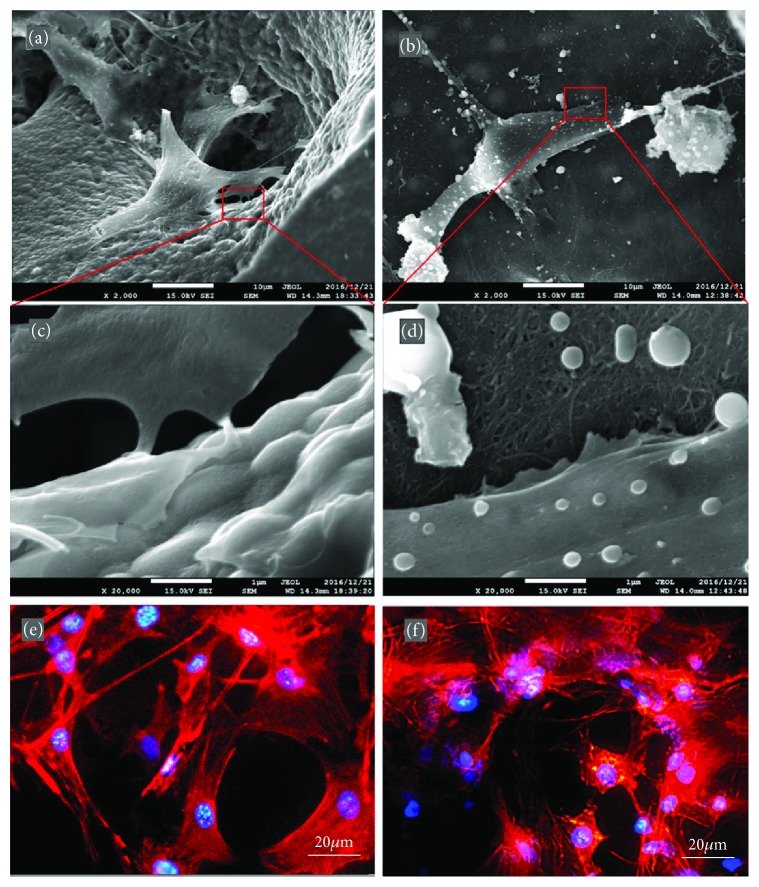
SEM and fluorescence microscope images of MC3T3-E1 cells on IP-CHA and CNTp scaffolds. (a, c, and e) On IP-CHA. (b, d, and f) On CNTp. (a, b, c, and d) IP-CHA and (b and d) CNTp. Magnification: 2000x for (a and c), 20,000x for (c and d). (e and f) Cells were labeled for actin filaments (red) and nucleus (blue) (reprinted from Tanaka et al. [159] with permission).

In another study, a three-dimensional (3D) scaffold for bone recognition was fabricated using MWNTs [159]. MWNT networks were prepared with polyacrylonitrile (PAN) followed by the addition and mixing of polymethyl-methacrylate (PMMA) microspheres to fabricate microporous structures of PMAA, PAN, and MWNTs.  Figure 7 shows SEM and fluorescent microscope images of MC3T3-E1 cells that had spread on the fabricated structures. Figures 7(a), 7(c), and 7(e) are images of IP-CHA, an established commercial product used as the control. Figures 7(b), 7(d), and 7(f) depict the results from similar experiments with the aforementioned CNTp nanoporous scaffolds. SEM clearly revealed the adherence of cells to both scaffold surfaces. The authors described the advantages of CNTp scaffolds based on various characterization experiments.

In cell studies, microscale elevation changes are expected. In particular, when cells are mixed with CNT nanobioconjugates, the heights of the hybridized objects can exceed 20 microns. SEM has proved useful to discern this topography. However, SEM observation of the behavior of living cells with CNTs is difficult since the examination is typically carried out in vacuum. Although environmental and atmospheric pressure SEMs are available, there are various constraints to their use. It is anticipated that SPM will soon be amenable for the time-lapse observation of living cells with CNT nanobioconjugates. Then, optical microscopes will truly be competitive observation tools.

## 6. CNT Nanobioconjugates as Sharp Probes

The small diameters of CNTs could be well suited to their use as sharp probes. The use of CNTs as SPM probes is one of the important scanning applications [136, 220–230].

Improvement of AFM resolution using a CNT tip is the most typical approach. In many applications, a single CNT is attached to the top of the usual AFM tip. In one study, an MWNT tip was used for AFM as well as scanning tunneling microscopy (STM) [220]. Hafner et al. demonstrated the direct growth of MWNT on an SPM tip [221]. Stevens et al. applied an MWNT AFM tip to observe 2 nm diameter iridium particles on mica surfaces in aqueous solutions [225].

Another important application is the use of CNTs as an “injector” for living cells. The attachment of cargo for drug delivery via disulfide bonding has been proposed [136]. The authors fabricated an AFM tip with an MWNT using SEM with a manipulator. Then, hybrids of quantum dot (QD) and streptavidin were attached on the MWNT surfaces using a crosslinker containing disulfide bonding. The functionalized AFM tip was injected into HeLa cells under AFM guidance, and QD was spontaneously released.

Various uses of CNTs as a sharp SPM probe can be envisioned. Single-cell surgery using drug delivery CNT probes is of one use. If induced pluripotent stem cells could be managed using this approach, it would be a fundamentally important advancement in medicine. Such applications demand the establishment of means of mass production of SPM probes with a CNT tip. CNT tips are currently handmade. Thus, for now, the accumulation of huge experimental data is difficult.

## 7. Characterization of CNT Nanobioconjugates by Scanning Techniques

Electrical properties of CNTs are defined due to their chirality. STM is a powerful tool to investigate the electrical properties of individual CNTs [231–234]. In one study, hybrids of DNA and MWNTs were observed by STM and scanning tunneling spectroscopy (STS) profiles were affected by the attachment of DNA molecules to MWNTs [231]. STS of SWNTs wrapped with ssDNA can be affected by DNA sequences [234]. A theoretical model has been proposed to explain the experimental STM and STS results [233].

**Figure 8:**
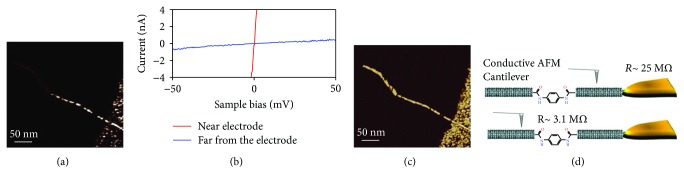
Representative conductive AFM image of a MTJ formed using PPD as the molecular linker and interfaced to a macroscopic metal electrode. (b) Representative I–V curves recorded at selected points across the MTJ: red line for measurements in close proximity to the macroscopic electrode and blue line for measurements at the far end from the macroscopic electrode. (c) Phase AFM image of the MTJ shown in (a). (d) Schematic representation of the conductive AFM measurements on the MTJs (reprinted from Zhu et al. [237] with permission).

Another scanning technique to investigate electrical properties of CNT nanobioconjugates is conductive AFM [235–237]. In one study, molecular transport junctions (MTJs) were fabricated. Two metallic SWNTs wrapped with DNA molecules were connected with p-phenylenediamine (PPD), and the resistance of the MTJs was measured by conductive AFM (Figure 8) [237].

The electrical properties of CNTs can be studied using two approaches. The precise assessment of the electrical properties of CNTs can be done in vacuum. Conversely, for biological applications, experiments should be carried out in liquids or in air/gas. In these cases, water and other molecules strongly affect the data. Several researchers focused on the effects of water molecules on physicochemical properties of CNTs [238–240]. If the samples include ions and other chemicals, which are commonly used for biological experiments, the effects can be markedly more complex.

## 8. Structures and Mechanical Properties Studied by AFM

AFM is the most convenient SPM for the standard characterization of CNT nanobioconjugates. Many researchers observed 3D structures of CNTs by AFM, and diverse lengths and widths of various types of CNT nanobioconjugates have been reported [69, 241–252]. Biochemical reactions, such as the interaction between protein and DNA molecules on CNT surfaces, were studied by AFM and other scanning techniques [232, 245, 253–264].

An impressive structural study using AFM described various ssDNA molecules on CNT surfaces [264]. Using the phase imaging mode, the authors found that the helical pitch of d(GT)_30_ was approximately 18 nm.

**Figure 9:**
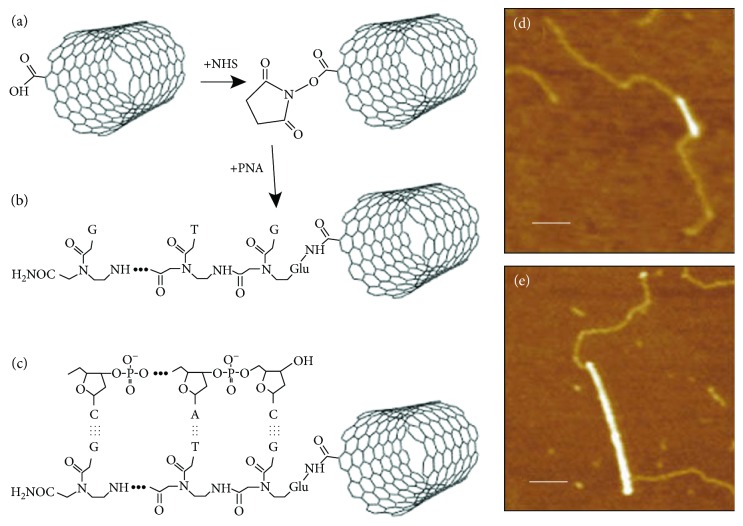
Hybridization of PNA attached SWNTs and DNA. (a and b) PNA was attached to an end of SWNT with N-hydroxysuccinimide (NHS) esters. (c) Hybridization of DNA with PNA attached SWNT. (d and e) AFM images of PNA-SWNTs. Bright lines indicate SWNTs. The paler strands represent bound DNA. Scale bars are 100 nm. Diameters of SWNTs were 0.9 nm and 1.6 nm in (d) and (e), respectively (reprinted from Williams et al. [263] with permission).

In another study, a peptide nucleic acid (PNA, NH_2_-Glu-GTGCTCATGGTG-CONH_2_) was fabricated and attached to the PNA at the ends of individual SWNTs. When ssDNA molecules having complementary sequences with DNA portions of PNA were reacted, the complementary DNA molecules avidly recognized the DNA regions. The hybridization was clearly confirmed by AFM observation (Figure 9) [263].

Force spectroscopy using AFM is a unique method to directly measure interactions between organic molecules and CNTs [232, 265, 266]. In one study, a DNA molecule was inserted into the the smooth inner pores of CNTs and then retracted to measure the frictional force between the DNA and the CNT [265]. DNA extraction from CNT pores occurred at a nearly constant force. In another study, a DNA molecule was peeled from an SWNT surface [232]. The peeling force of ssDNA from SWNTs was much greater than that from flat graphite. A recent study provided dynamic observations of CNT nanobioconjugates using high-speed AFM [267].

Although there are numerous SPM studies that have provided routine AFM pictures of CNT nanobioconjugates, the use of specific functions of SPMs, such as force measurements, has been very limited. Future studies will hopefully utilize the full SPM functional repertoire to study CNT nanobioconjugates. For example, various types of forces, including friction, electrostatic, acoustic, and magnetic forces, can be measured by specific AFM options. Typical SPM options include scanning near-field optical microscopy, scanning thermal microscopy, scanning electrochemical microscopy, scanning Kelvin probe force microscopy, scanning chemical potential microscopy, scanning ion conductance microscopy, and scanning capacitance microscopy.

## 9. Manipulation of CNT Nanobioconjugates by Scanning Techniques

Manipulation of CNTs by SEM is a popular tool. Attachment of CNTs on SPM probes is usually carried out this way [136, 220–222, 224–230]. In one study, single CNT was attached between two AFM probes under SEM observation [230]. Then, the two AFM tips were separated to directly measure the breaking force of the CNT. The authors estimated that the breaking force was 1.3 *μ*N.

AFM is advantageous to manipulate CNT nanobioconjugates in air or liquids. “Dragging” CNTs by an AFM tip was described. The CNTs were distorted and digested by manipulation with an AFM tip [268]. Other authors proposed DNA carriers using CNTs based on molecular dynamics calculation [269]. In their proposal, DNA molecules that are inserted into the inner pores of CNTs can be moved by CNT manipulation using an AFM tip.

The potential of single-cell surgery was mentioned earlier. Similarly, single-molecule surgery of nanobioconjugates with CNTs using the manipulation technique is also a challenging research target. Single-molecule surgery of CNT nanobioconjugates might be realized with a single SPM probe of CNT nanobioconjugates.

## 10. Approaches Using Mapping Methods Related Scanning Technique

One of the unique optical properties of CNTs is photoluminescence (PL) from CNTs. For example, Ito et al. combined PL measurements and SEM observation [270]. When (9, 4) SWNTs are excited with a laser wavelength of 730 nm, the SWNTs photoluminesce at 1130 nm. Excitation and emission wavelengths vary among CNTs with different chiralities. Furthermore, the emission wavelength and PL intensity fluctuate in a sensitive response to oxidation/reduction and other factors. By using PL measurements, various applications of CNT nanobioconjugates, such as nanobiosensors, are available. For PL measurements, a “PL map” can be obtained. Usually, the *x*- and *y*-axis of a PL map is the emission and excitation wavelength, respectively. Although SEM and SPM spatially scan the sample surface, PL reveals wavelength scanning. PL measurements by a variety of scanning techniques would provide rich information about CNT nanobioconjugates.

In a recent study, AFM infrared spectroscopy was used to study the morphological and optical properties of CNTs [271]. This new scanning technique provides spatial information of CNT nanobioconjugates.

Mapping techniques, such as PL mapping, and scanning techniques, such as SPM and SEM, have been independently used in various research fields. However, for the study of CNT nanobioconjugates, we believe that the combined use of the two techniques will be a boon to discovery. In particular, to understand the mechanisms of several unique responses of CNTs, the combined structural and physicochemical information would be valuable.

## 11. Conclusion

In this paper, the contributions of scanning techniques to studies of CNT nanobioconjugates have been summarized and future prospects discussed. Research subjects are categorized based on CNT applications not on the types of scanning methods. In addition, the possibility of a combination of mapping techniques and scanning techniques is also described. We hope that this review article informs future studies in this field.
